# Supplementary material for: 3-Hydroxytanshinone Inhibits the Activity of Hypoxia-Inducible Factor 1-α by Interfering with the Function of α-Enolase in the Glycolytic Pathway
Source: Molecules. 2024 May 9;29(10):2218. doi: 10.3390/molecules29102218 (PMC11123766; doi:10.3390/molecules29102218)
Supplement: Supplementary file 1 [file molecules-29-02218-s001.zip › molecules-2974696-supplementary.pdf]

molecules

3-Hydroxytanshinone Inhibits the Activity of Hypoxia-Inducible Factor 1-α by Interfering with the Function of α-Enolase in the Glycolytic Pathway

Tae Hyun Son [1,2,†], Shin-Hye Kim [2,†], Hye-Lim Shin [2,3], Dongsoo Kim [2], Hwan Gyu Kim [3], Yongseok Choi [1,*] and Sik-Won Choi [2,*]

[1] School of Life Sciences and Biotechnology, Korea University, Seoul 02841, Republic of Korea; snoopyegg@korea.ac.kr
[2] Forest Biomaterials Research Center, National Institute of Forest Science (NIFoS), Jinju 52817, Republic of Korea; black7a@korea.kr (S.-H.K.); hlims0901@korea.kr (H.-L.S.); skimds@korea.kr (D.K.)
[3] Department of Biological Sciences, Jeonbuk National University, Jeonju 54896, Republic of Korea; hgkim@jbnu.ac.kr
* Correspondence: ychoi@korea.ac.kr (Y.C.); superwon@korea.kr (S.-W.C.); Tel.: +82-2-3290-3426 (Y.C.); +82-55-760-5093 (S.-W.C.); Fax: +82-55-759-8432 (S.-W.C.)
† These authors contributed equally to this work.

Citation: Son, T.H.; Kim, S.-H.; Shin, H.-L.; Kim, D.; Kim, H.G.; Choi, Y.; Choi, S.-W. 3-Hydroxytanshinone Inhibits the Activity of Hypoxia-Inducible Factor 1-α by Interfering with the Function of α-Enolase in the Glycolytic Pathway. *Molecules* **2024**, *29*, 2218. https://doi.org/10.3390/molecules29102218

Academic Editors: Luciana Scotti and Marcus Tullius Scotti

Received: 5 April 2024
Revised: 29 April 2024
Accepted: 7 May 2024
Published: 9 May 2024

Copyright: © 2024 by the authors. Licensee MDPI, Basel, Switzerland. This article is an open access article distributed under the terms and conditions of the Creative Commons Attribution (CC BY) license (https://creativecommons.org/licenses/by/4.0/).

3-Hydroxytanshinone

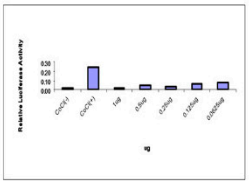

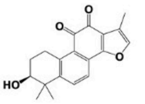

C19H18O4

Mol. Wt.: 310.34

(S)-1,6,6-trimethyl-10,11-dioxo-6,7,8,9,10,11-hexahydrophenanthro[1,2-b]furan-7-yl propionate

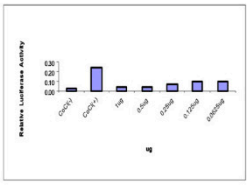

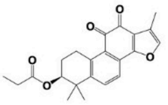

C22H22O5

Mol. Wt.: 366.41

(S)-1,6,6-trimethyl-10,11-dioxo-6,7,8,9,10,11-hexahydrophenanthro[1,2-b]furan-7-yl benzoate

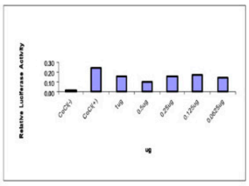

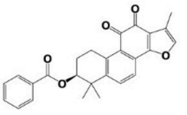

C26H22O5

Mol. Wt.: 414.45

(S)-7-(2-hydroxyethoxy)-1,6,6-trimethyl-6,7,8,9-tetrahydrophenanthro[1,2-b]furan-10,11-dione

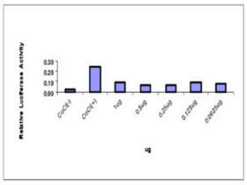

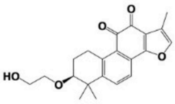

C21H22O5

Mol. Wt.: 354.4

Biotinylated-3-hydroxytanshinone

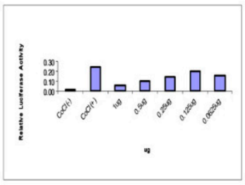

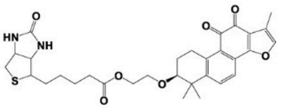

C31H36N2O7S

Mol. Wt.: 580.6

(S)-2-((1,6,6-trimethyl-10,11-dioxo-6,7,8,9,10,11-hexahydrophenanthro[1,2-b]furan-7-yl)oxy)ethyl 2-(3-(3-(trifluoromethyl)-3H-diazirin-3-yl)phenoxy)acetate

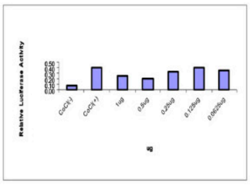

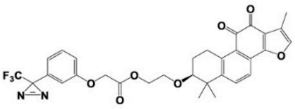

C31H27F3N2O7

Mol. Wt.: 596.55

6-(hydroxymethyl)-1,6-dimethyl-6,7,8,9-tetrahydrophenanthro[1,2-b]furan-10,11-dione

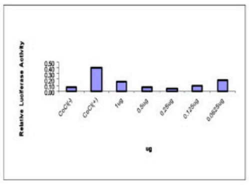

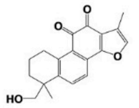

C19H18O4

Mol. Wt.: 310.34

(1,6-dimethyl-10,11-dioxo-6,7,8,9,10,11-hexahydrophenanthro[1,2-b]furan-6-yl)methyl 2-(2-hydroxyethoxy)acetate

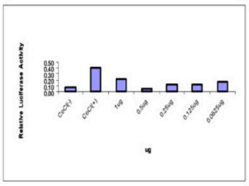

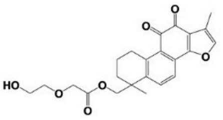

C23H24O7

Mol. Wt.: 412.43

2-(2-((1,6-dimethyl-10,11-dioxo-6,7,8,9,10,11-hexahydrophenanthro[1,2-b]furan-6-yl)methoxy)-2-oxoethoxy)ethyl 2-(3-(3-(trifluoromethyl)-3H-diazirin-3-yl)phenoxy)acetate

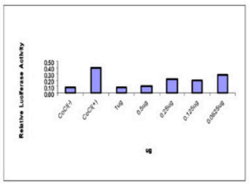

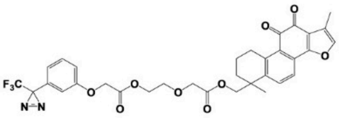

C33H29F3N2O9

Mol. Wt.: 654.59

2-(2-((1,6-dimethyl-10,11-dioxo-6,7,8,9,10,11-hexahydrophenanthro[1,2-b]furan-6-yl)methoxy)-2-oxoethoxy)ethyl 5-(2-oxohexahydro-1H-thieno[3,4-d]imidazol-4-yl)pentanoate

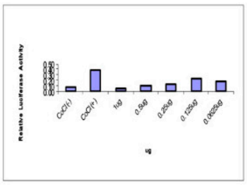

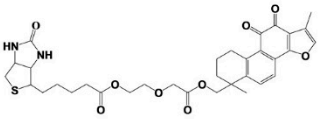

C33H38N2O9S

Mol. Wt.: 638.73

Supplementary Figure S1. Tanshinone derivates regulates HIF-1α activity. A mixture (3:1) of two stable cell lines (HeLa-hypoxia response element [HRE]-firefly luciferase [FL] and HeLa-CMV-Renilla luciferase [RL]) was plated into 96-well plates at a density of 4000 cells per well. The cells were incubated for 16 hours in the presence of $CoCl_2$ with the indicated concentrations of tanshinone derivates. Luciferase activity was determined. Activity is expressed as fold induction relative to the activity in the absence of $CoCl_2$. HeLa-CMV-RL activity was used to normalize luciferase activity.
